# CLIC6 and ANLN: novel exosome-related prognostic markers and therapeutic targets in lung adenocarcinoma

**DOI:** 10.3389/fimmu.2026.1756058

**Published:** 2026-03-23

**Authors:** Yajun Miao, Tao Li, Rong Li, Yufei Liu

**Affiliations:** 1 Department of Oncology, The First People's Hospital of Nantong, Nantong, China; 2 Department of Medical Oncology, Affiliated Tumor Hospital of Nantong University, Nantong, China; 3 Department of Medical Oncology, Taikang Xianlin Drum Tower Hospital, Nanjing, China; 4 Department of Medical Oncology, Ma’anshan General Hospital of Ronger-Duree Healthcare, Ma’anshan, China; 5 Department of Medical Oncology, Jiangsu Cancer Hospital and Jiangsu Institute of Cancer Prevention and Treatment, The Affiliated Cancer Hospital of Nanjing Medical University, Nanjing, China

**Keywords:** exosomes, immunity, lung adenocarcinoma, prognosis, ScRNA-seq, TIDE

## Abstract

**Background:**

Exosomes can promote tumor development and regulate tumor immune responses, making them of significant value in Lung Adenocarcinoma (LUAD) management. In-depth exploration of exosome-related genes in LUAD is of great significance for expanding LUAD clinical treatment options.

**Methods:**

Data from The Cancer Genome Atlas (TCGA) and Gene Expression Omnibus (GEO) were analyzed. Differential expression analysis (limma package), consensus clustering (ConsensusClusterPlus), and Least Absolute Shrinkage and Selection Operator (LASSO) regression (glmnet package) were used to build a prognostic model. Immune infiltration was assessed with TIMER, MCPcounter, and single-sample gene set enrichment analysis (ssGSEA). Tumor Immune Dysfunction and Exclusion (TIDE) algorithm evaluated immunotherapy response. Single-cell RNA sequencing (scRNA-seq) data were processed using Seurat. LUAD cell lines (A549, NCI-H838) were used for quantitative real-time PCR (qRT-PCR), Cell Counting Kit-8 (CCK-8), Transwell, and wound-healing assays.

**Results:**

Four genes associated with exosomes were identified as key genes significantly influencing LUAD prognosis, namely CLIC6, ANLN, FAM83A, and RHOV. A LUAD prognosis model was constructed based on these genes, and the ROC curve confirmed the model’s excellent predictive performance. Immune infiltration analysis revealed immune cell infiltration differences between low- and high-RiskScore groups in LUAD, with significant differences in infiltration observed between groups for cells including eosinophils, T cells, myeloid dendritic cells, and B lineage (*P* < 0.05). CD274, PDCD1 and LAG3 were highly expressed in the high-risk group of LUAD (*P* < 0.01). Exosome-related pathways were significantly enriched in epithelial cells and monocyte/macrophage cells. Single-cell analysis reveals that CLIC6 exhibits higher expression levels in epithelial cells. ANLN regulates the malignant phenotype of LUAD cell lines.

**Conclusion:**

A four-gene exosome-related signature was identified that can effectively separate LUAD patients into risk subgroups having distinct immune characteristics and immunotherapeutic benefits. Which occurred when ANLN was validated as an oncogene, coupled to the model being significantly associated with immune evasion mechanisms, suggests these biomarkers could enhance prognostic assessment and allow clinicians to identify patients who may be less likely to respond to immunotherapy, thereby informing personalized treatment strategies.

## Introduction

Based on pathological classification, Lung Adenocarcinoma (LUAD) has become the most common subtype of lung cancer (1–3), with an increasing incidence rate, especially among young women and smokers (4, 5). In recent years, LUAD treatment has evolved beyond traditional surgical, chemotherapy, and radiotherapy approaches to include new strategies such as targeted therapy, immunotherapy, and personalized treatment (6, 7). However, due to issues such as high heterogeneity and drug resistance, the overall survival rate for LUAD remains low, and mortality rates remain high (8). Currently, treatment strategies for LUAD are evolving toward greater diversity and personalization, with comprehensive treatment approaches combining multiple modalities emerging as a new trend (9, 10). Identifying LUAD-related prognostic genes and establishing prognostic models are crucial foundations for achieving personalized treatment and also facilitate further research into LUAD.

Exosomes are small vesicles secreted by a variety of cell types, containing biological signals such as proteins, mRNA, miRNA, and DNA (11, 12). When they act on recipient cells, they can cause changes in the morphology and function of the corresponding cells (13–15). Therefore, exosomes can serve as key mediators of intercellular communication (16). Increasing research indicates that proteins and nucleic acids in exosomes can serve as biomarkers for tumor diagnosis and prognosis, possessing significant clinical value (17, 18). High levels of miR-20b-5p are found in exosomes produced by non-small cell lung cancer (NSCLC) cells compared to exosomes from normal cells. This microRNA promotes cancer cell proliferation by downregulating the type 2 transforming growth factor-beta (TGF-β) receptor and enhances cancer cell invasion and the complex tumor microenvironment (19). Additionally, studies have shown that exosomes derived from M2-type macrophages significantly increase NSCLC cell migration and invasion by delivering integrin αVβ3; when integrin is blocked, the effects of M2-type macrophage-mediated NSCLC cell invasion and migration are significantly reduced (20). In terms of immune regulation, related studies have found that tumor cell-secreted exosomes can induce macrophage polarization toward an immunosuppressive phenotype through metabolic reprogramming mechanisms (21). Exosomes significantly accelerate the glycolytic process of macrophages by activating the NF-κB-dependent glycolytic pathway, promoting lactate production. Exogenous lactate, in turn, directly activates the NF-κβ signaling pathway, forming a positive feedback loop to sustain a high expression of PD-L1 on the surface of macrophages, ultimately promoting the formation of an immunosuppressive pre-metastatic niche (21, 22). This undoubtedly provides important insights into the mechanisms of immune regulation in lung cancer progression. However, the specific exosome-related genes that drive LUAD progression and modulate anti-tumor immunity remain to be elucidated.

This study introduces an innovative approach by systematically integrating exosome-associated gene profiling with single-cell transcriptomic and tumor immune microenvironment analysis in LUAD. The research was designed to identify critical molecular features linked to exosomes, construct a comprehensive prognostic framework, and explore its association with immunological characteristics and therapy response. The potential significance of this work lies in providing a novel multi-dimensional tool for patient stratification and offering foundational insights for advancing personalized immunotherapy in LUAD.

## Materials and methods

### Data acquisition

The TCGA (https://portal.gdc.cancer.gov) database was accessed to obtain gene expression data and clinical data of LUAD samples. After deleting patients without complete survival time and status or survival time shorter than 0 day, the RNA-seq expression profiles of 500 tumor samples and 59 control samples were converted to FPKM format and log2-transformed. The GSE31210 dataset with 226 tumor samples was downloaded from GEO. In this study, the TCGA-LUAD dataset and GSE31210 dataset served as the training and independent validation sets, respectively. GSE149655 from the GEO database was obtained. Based on the provided file containing sample information corresponding to each cell, we selected tumor lesions and related control samples, including 2 tumor lesion samples and 2 control samples. In this study, 2,035 lung cancer-derived exosomes were obtained from the exocarta database (http://www.exocarta.org/) (23). Detailed information on these genes is provided in **Supplementary Table S1**.

### LUAD subtype clustering

Under the criteria of |log 2 (FC)| > log2(2) and adjusted *p* < 0.05, differentially expressed genes (DEGs) between NSCLC and control samples were filtered by the limma package (24). The intersection of DEGs and genes related to exosomes was taken to obtain DEGs related to exosomes. Subsequently, the ConsensusClusterPlus package (25) was used to cluster the tumor samples, specifically with clusterAlg = “hc”, distance = “pearson”, and 500 sampling repetitions, each with an 80% sampling proportion. According to cumulative distribution function (CDF), we determined the optimal number of clusters.

### LUAD risk assessment model construction

Following univariate Cox regression on DEGs from exosome-related LUAD subtypes, LASSO-Cox regression was performed using the glmnet package (26) with 10-fold cross-validation to select the optimal penalty parameter λ (lambda.min), thereby shrinking coefficients to prevent overfitting. Subsequently, stepwise regression based on the Akaike Information Criterion (AIC) was applied using the stepAIC function from the MASS package to further refine the prognostic model. Subsequently, a LUAD prognosis risk assessment model was developed as follow:


$$Riskscore=\sum \beta_{i\;}*\text{expression i}$$

(i refers to the level of gene expression, and β is the Cox regression coefficient of the gene).

Following stratification of patients into high- and low-risk groups according to the median RiskScore, survival outcomes were compared using Kaplan–Meier analysis and significance of differences was compared by log-rank test. Employing the R software package timeROC (27), ROC curve was plotted to assess the model reliability in prognostic and survival prediction.

### Immune infiltration analysis of LUAD

This study used the TIMER algorithm to compute immune cell scores for the two risk groups in TCGA-LUAD. The MCPcounter package (28) and ssGSEA were used to calculate the differences in immune cell infiltration in LUAD samples with different RiskScores.

### Immunotherapy response assessment

Immunotherapy response of LUAD was evaluated by the TIDE method. The TIDE score was computed by importing LUAD transcriptomic data into the TIDE website (http://tide.dfci.harvard.edu/), with a higher TIDE indicating less immunotherapy benefit.

### The scRNA-Seq analysis

This study used the Read10X function of the Seurat package (29) to read the data, retaining cells with a mitochondrial gene proportion <20% and a gene count between 200 and 7500. Data were standardized with the SCTransform function, followed by using the RunPCA function to conduct principal component analysis (PCA). The harmony package (30) was used to remove batch effects between different samples. Here, the first 30 PCs were used for UMAP dimensionality reduction. The FindNeighbors and FindClusters functions were used to cluster cell subpopulations (resolution=0.1). Finally, marker genes provided by the CellMarker2.0 database were used to annotate each cell type. Finally, to assess the enrichment activity of exosome-related pathways in tumor lesions as compared to adjacent non-tumor samples, the AUCell method (31) was used to calculate pathway activity scores at the single-cell level using single-cell transcriptomic data.

### Cell culture and transfection

DMEM with 10% FBS at 37 °C was employed to cultivate human cell lines BEAS-2B, A549, and NCI-H838 (ATCC, USA) in a humidified atmosphere containing 5% CO_2_. For gene silencing, cells were transfected with siRNA targeting the indicated genes or si-NC utilizing Lipofectamine 3000 reagent (Invitrogen, USA) in strict accordance with protocol. Here, we selected only the ANLN gene knockdown for subsequent functional validation experiments. The sequences of si-target gene were as follows: si-ANLN (GTGAAGAGAAATCTTGTACAAAA and GAGAAATCTTGTACAAAACCATC).

### Quantitative real-time PCR

Using the Trizol reagent, total RNA was isolated to synthesize cDNA with the Qiagen One-Step RT-PCR Kit (Qiagen GmbH, Germany). qRT-PCR was conducted on the ABI 7500 Real-Time PCR System (Thermo Fisher Scientific, USA) using SYBR Green Master Mix to evaluate relative gene expression. The relative mRNA levels were analyzed and normalized to GAPDH expression using the 2^−ΔΔct^ method. The primers were as follows:

CLIC6 (Forward: GACATCACCCTCTTCGTCAAGG; Reverse: CTTTTCAGGTCCACTGTGGTCAC).

ANLN (Forward: CAGACAGTTCCATCCAAGGGAG; Reverse: CTTGACAACGCTCTCCAAAGCG).

FAM83A (Forward: ATCCAGCGCCACTGTGTACTTC; Reverse: CCGTGAACACATCCATCAGGATG).

RHOV (Forward: ACTGCGCTGGACACCTTCTCTG; Reverse: CACGCCAGGAAGACATCGGTAT).

GAPDH (Forward: GTCTCCTCTGACTTCAACAGCG; Reverse: ACCACCCTGTTGCTGTAGCCAA).

### Cell proliferation assay

CCK-8 assay (Dojindo Laboratories, Japan) was used for measuring cell viability. Briefly, cells were seeded into 96-well plates (10 μL per well) and cultured for the indicated times. The absorbance at 450 nm was measured using a microplate reader.

### Cell invasion assay

Upper Transwell chambers (Corning, USA) were pre-coated with Matrigel and added with cells, while the lower chamber contained medium with 10% FBS. After 48-h incubation at 37 °C, invaded cells were fixed with 4% paraformaldehyde and colored by crystal violet for 10 min, whereas non-invaded cells were removed from the upper surface. The invaded cells was quantified with an inverted microscope.

### Wound healing assay

Cells were plated into 6-well plates to full confluence. A linear scratch was produced across the monolayer using a sterile pipette tip. Detached cells were gently washed away with PBS, and fresh serum-free medium was added. Images were captured at 0 h and 48 h using a microscope, and the wound closure rate was calculated as: (initial wound width – remaining wound width)/initial wound width × 100%.

### Statistical analysis

All statistical analyses were performed using R software (4.3.1) and GraphPad Prism 10.6.1. The differential expression analysis between tumor and normal samples was performed using the limma package. The differences in immune cell infiltration and checkpoint gene expression between risk groups were evaluated using the Wilcoxon signed-rank test. For comparisons in two independent groups, unless the assumptions of normality and homogeneity of variance were met, Welch’s unpaired t-test was employed. If those assumptions met the standard, unpaired Student’s t-test was used. In instances where there were more than two groups involved, one-way analysis of variance (ANOVA) was conducted. Tukey’s *post-hoc* test for multiple comparisons was used where appropriate. The data for all the *in vitro* experiments were represented as mean ± standard deviation (SD) from three independent replicates. In the study, a *p*-value less than 0.05 was considered to be statistically significant.

## Results

### Screening of LUAD exosome-related genes

**Figure 1:**
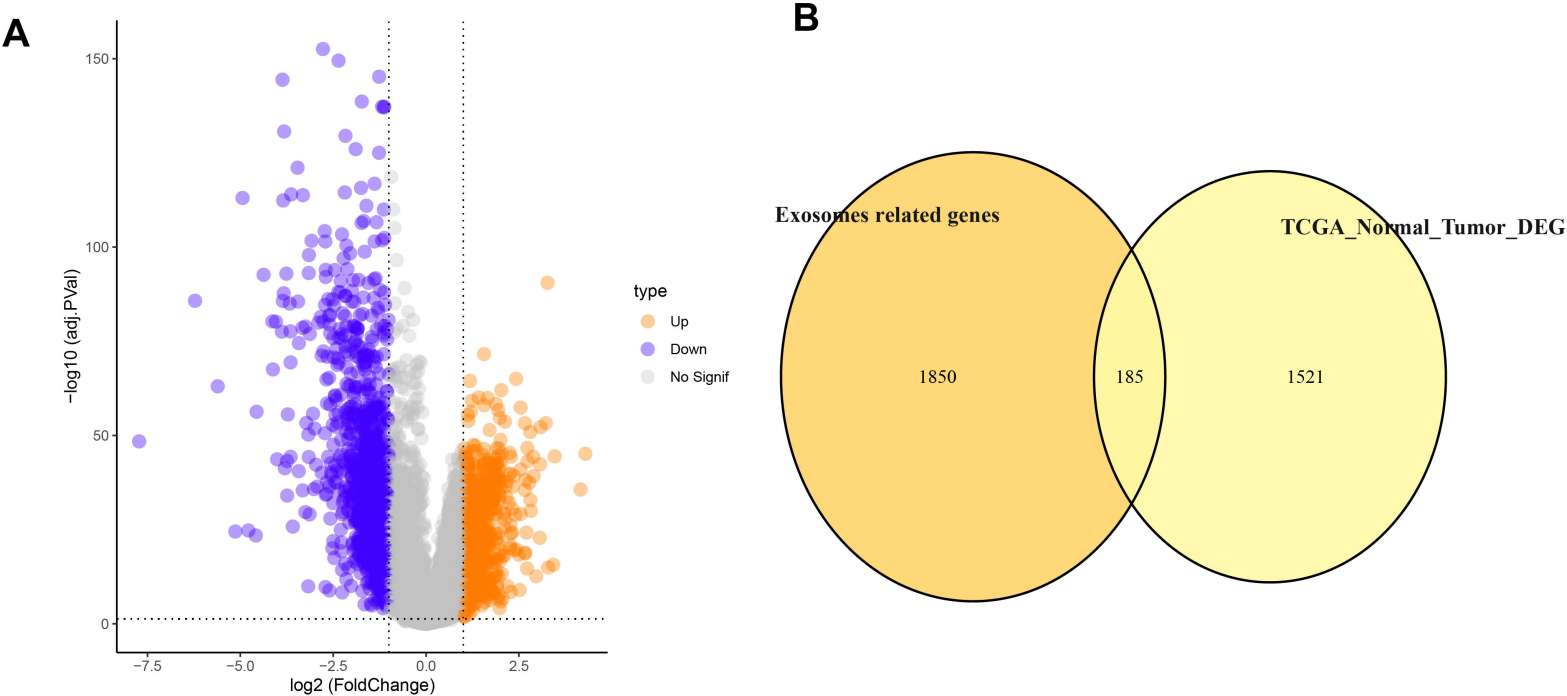
Differential expression analysis of LUAD. **(A)** Volcano plot of DEGs between TCGA-LUAD tumor samples and control samples. **(B)** The intersection between DEGs and exosome-associated genes in Venn diagram.

This study identified DEGs between TCGA-LUAD tumor samples and control samples, and further intersected them with exosome-related genes, obtaining a total of 185 LUAD exosome-related DEGs (**Figures 1A, B**).

### Molecular subtyping of LUAD based on exosome-related genes

**Figure 2:**
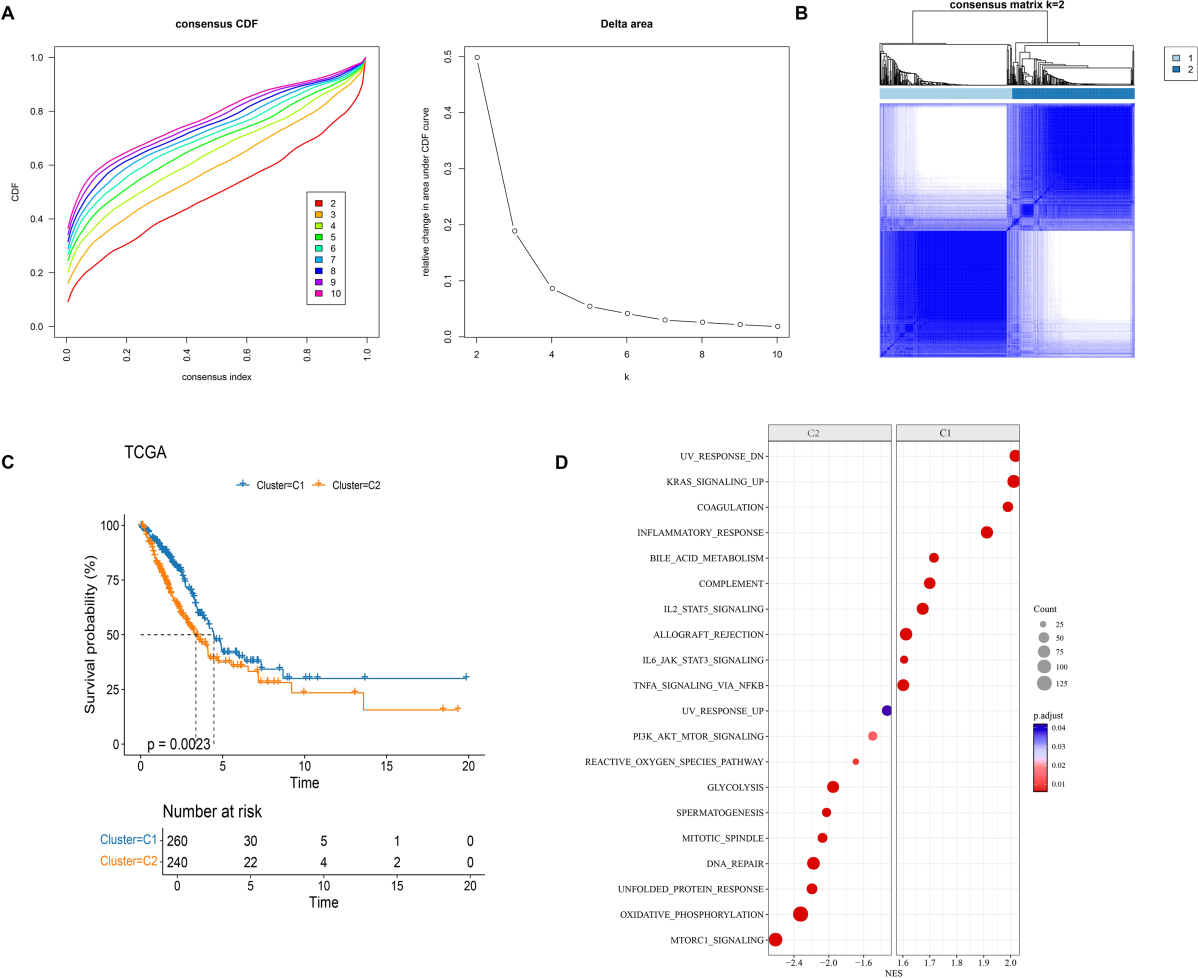
Consensus clustering of LUAD samples. **(A)** CDF curve and CDF Delta area curve of TCGA-LUAD samples. **(B)** Clustering heat map of TCGA-LUAD samples at consensus k=2. **(C)** KM curve of the overall survival (OS) prognosis relationship between the two subtypes in TCGA-LUAD. **(D)** Pathway differences between subtypes in GSEA analysis.

Using these 185 genes, this study employed the ConsensusClusterPlus method to perform cluster analysis on samples from the TCGA-LUAD dataset. By analyzing the CDF and CDF Delta area curve, the cluster number was set to 2, the clustering results exhibited high stability (**Figure 2A**). Therefore, this study determined the optimal number of clusters to be k=2, thereby classifying the samples into two molecular subtypes (C1 and C2) (**Figure 2B**). Two molecular subtypes showed significant prognostic differences, with the C1 exhibiting a better prognosis than C2 (**Figure 2C**). Enrichment analysis revealed that the C1 subtype was notably enriched in signaling pathways such as KRAS_SIGNALING_UP, COAGULATION, and INFLAMMATORY_RESPONSE, while the C2 subtype was significantly enriched in pathways including DNA_REPAIR, OXIDATIVE_PHOSPHORYLATION, MTORC1_SIGNALING, and GLYCOLYSIS (**Figure 2D**).

### Construction of a LUAD prognosis model

**Figure 3:**
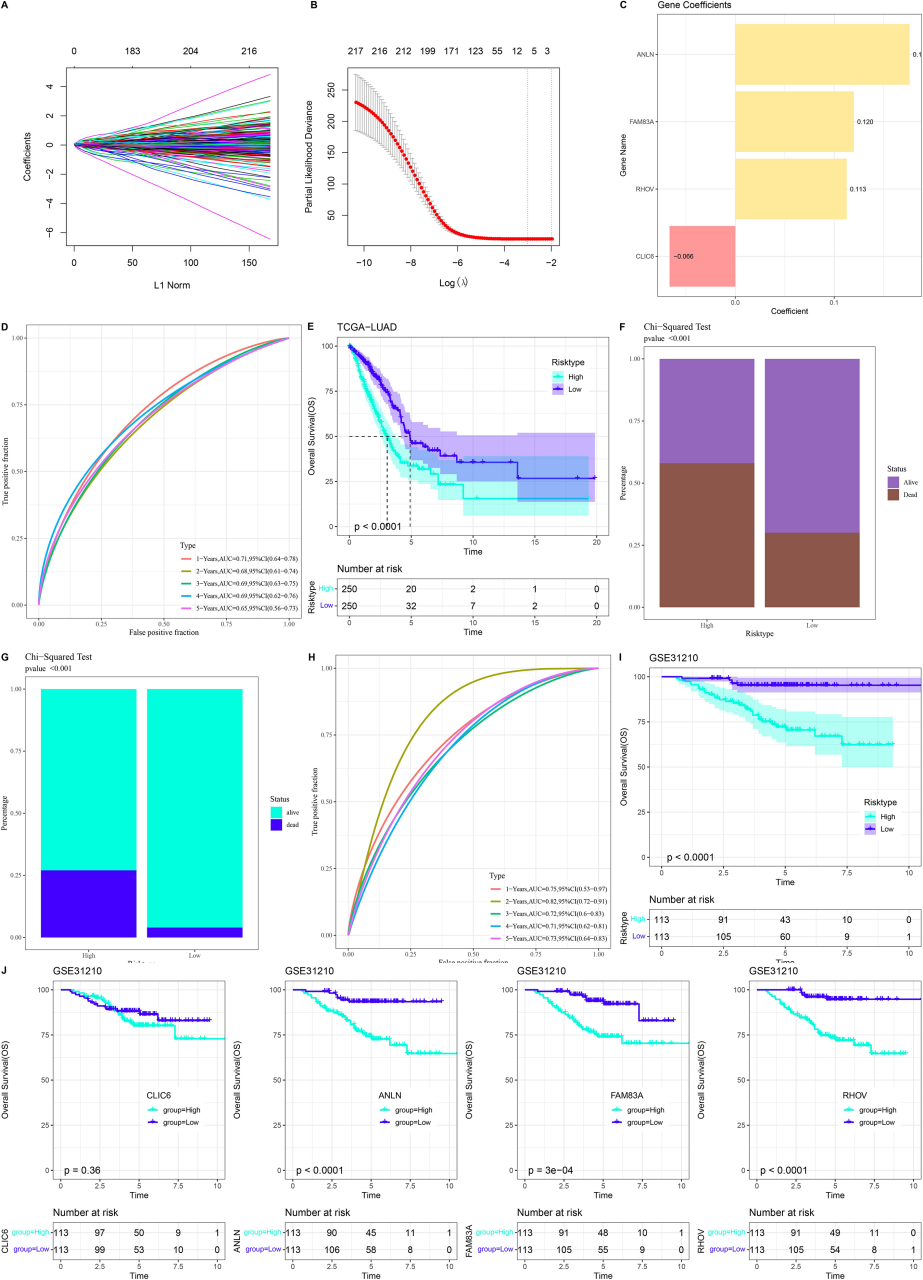
Development and evaluation of the LUAD prognostic model. **(A, B)** Distribution of gene coefficients generated with log(λ) in the LASSO model and the LASSO coefficient spectrum. **(C)** Risk coefficients of prognostic-related genes in the prognostic model in TCGA-LUAD. **(D, E)** ROC curves and survival curves evaluating the reliability of the LUAD prognostic model in TCGA-LUAD. **(F)** Proportion of deceased patients in the high- and low-risk groups in TCGA-LUAD. **(G)** Proportion of deceased patients in the high- and low-risk groups in the GSE31210 dataset. **(H, I)** ROC curves and survival curves for the GSE31210 dataset to assess the reliability of the prognostic model. **(J)** KM curves for the high- and low-expression groups of TCGA prognostic-related genes.

DEGs between LUAD subtypes were subjected to univariate COX regression analysis and LASSO regression analysis to develop a risk model, with 10-fold cross-validation to improve the generalization abilities of the model (**Figures 3A, B**). Through further stepwise multivariate regression analysis, this study identified four genes as significantly influencing LUAD prognosis and constructed a prognostic assessment model: Riskscore = -0.066 × CLIC6 + 0.176 × ANLN + 0.12 × FAM83A + 0.113 × RHOV (**Figure 3C**). This study classified LUAD patients into low-risk and high-risk groups based on Riskscore, and the ROC curve confirmed the reliability of the model in predicting 1- to 5-year survival outcomes for patients, with AUC values exceeding 0.6 (**Figure 3D**). Survival analysis results showed that LUAD patients with high Riskscore had significantly lower survival rates than those with low Riskscore, and the high Riskscore group had a higher number of deaths (**Figures 3E, F**). This study was validated in the GSE31210 dataset, yielding the same conclusions: LUAD patients with high Riskscore had more deaths and poorer prognosis, and the model accurately predicted patient survival outcomes over 1 to 5 years, with AUC values exceeding 0.6 on the ROC curve (**Figures 3G–I**). The present research assessed the importance of each gene’s impact on LUAD prognosis, revealing that LUAD samples with high expression of the ANLN, FAM83A, and RHOV genes had significantly poorer prognosis (**Figure 3J**).

### Assessment of immune infiltration levels in low- and high-risk groups of LUAD

**Figure 4:**
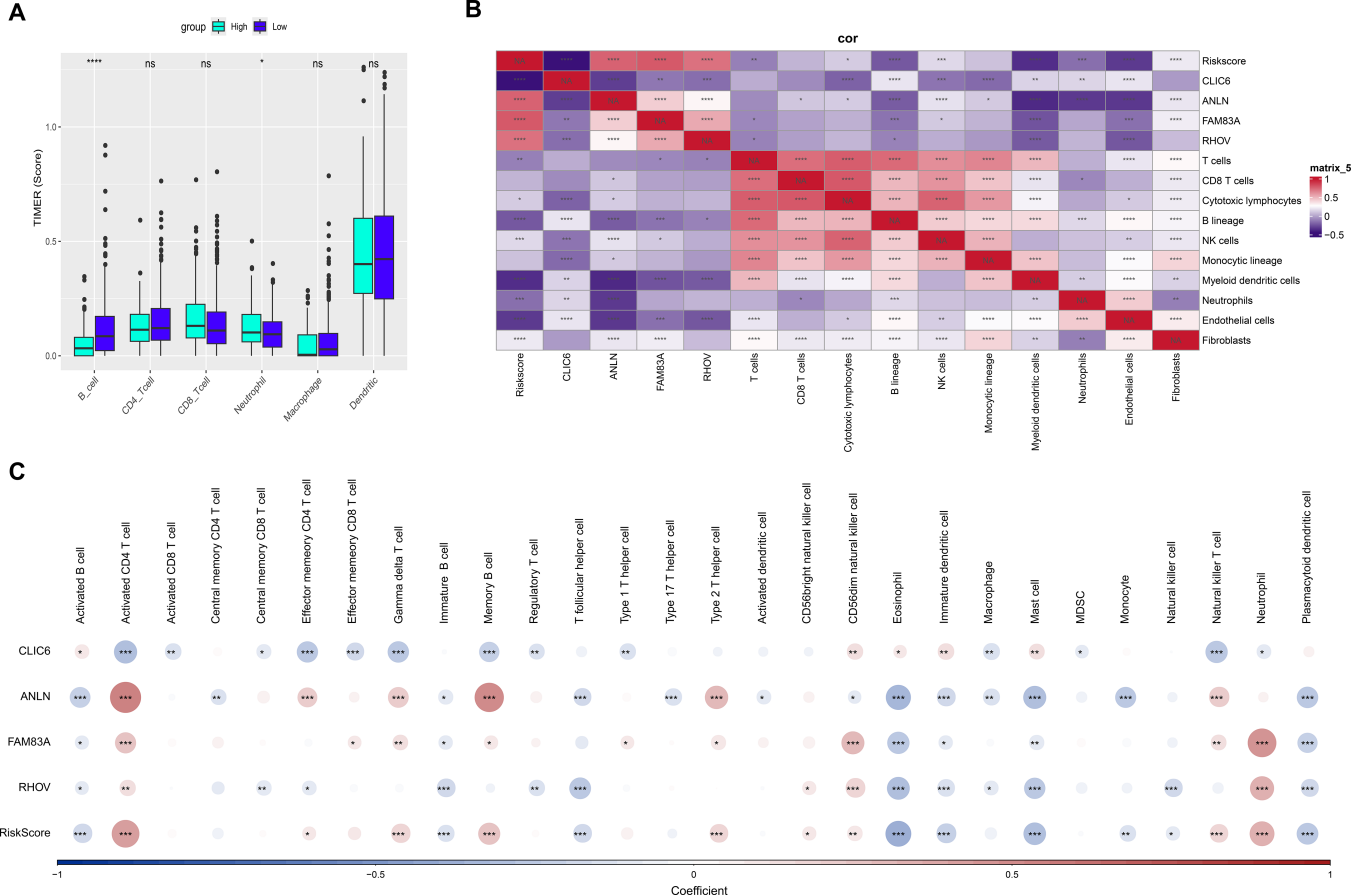
immune microenvironment of LUAD assessed by the prognostic model. TIMER **(A)**, MCP-counter **(B)**, and ssGSEA **(C)** analyzed the differences in immune cell infiltration between high- and low-risk LUAD groups. ns means *p* > 0.05; * *p* < 0.05, ** *p* < 0.01, *** *p* < 0.001, and **** *p* < 0.0001.

This study calculated the immune cell scores of TCGA-LUAD in different groups. It was found that neutrophils had higher immune-related scores in the low-risk group, which was statistically significant (**Figure 4A**). Additionally, myeloid dendritic cells, T cells, B lineage showed a significant negative correlation with Riskscore (**Figure 4B**). This study analyzed the correlation between Riskscore and ssGSEA immune cell scores, finding that eosinophils, mast cells, and plasmacytoid dendritic cells showed a significant negative correlation with Riskscore (**Figure 4C**).

### Immune response assessment in LUAD

**Figure 5:**
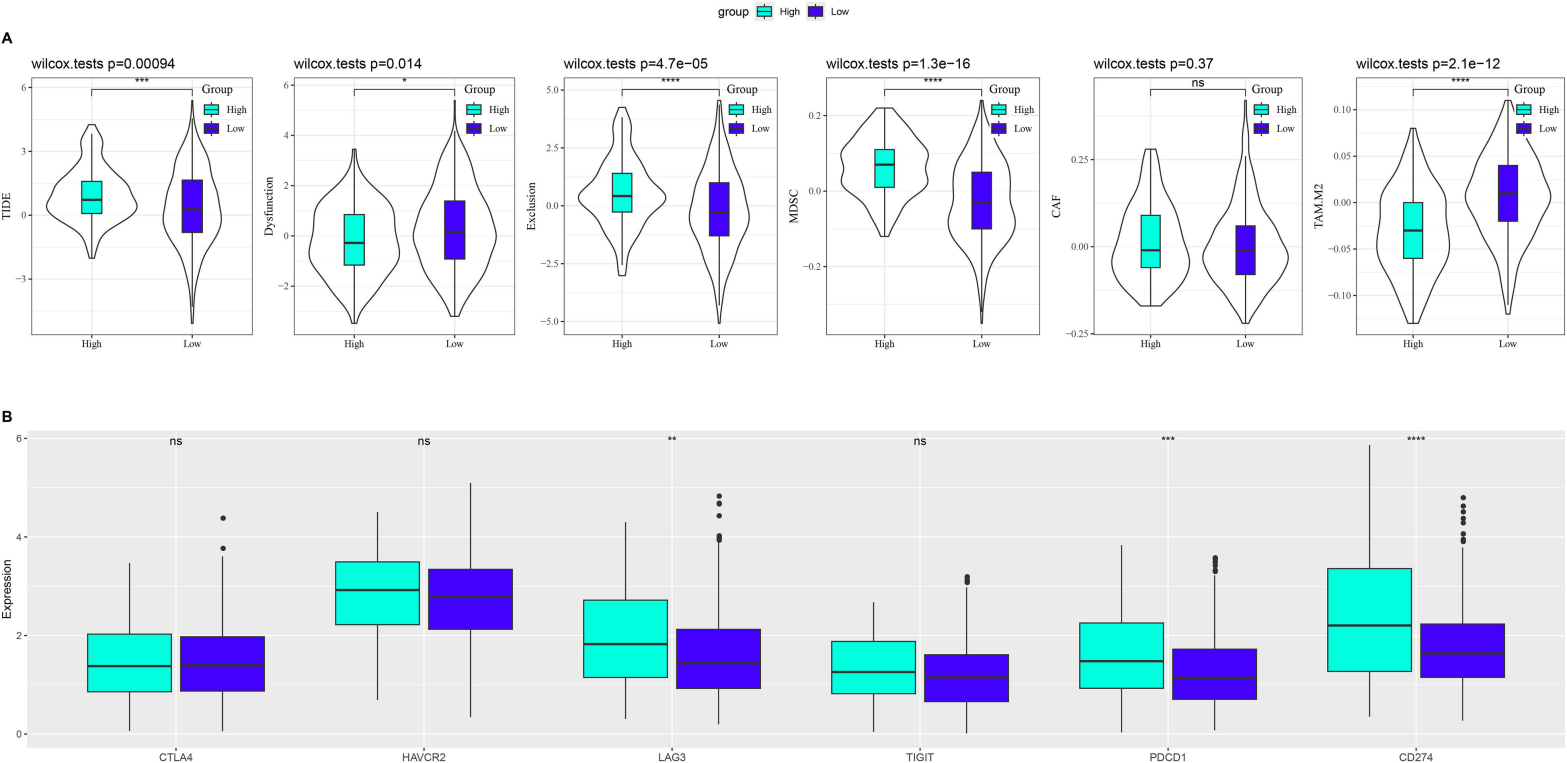
Analysis of immune therapy response in LUAD. **(A)** Differences in TIDE analysis results between LUAD groups with high and low risk. **(B)** Differences in immune checkpoint gene expression between the two risk LUAD groups. ns means p > 0.05; * p < 0.05, ** p < 0.01, *** p < 0.001, and **** p < 0.0001.

This study analyzed immunotherapy response differences between the two risk groups. First, we analyzed differences in immunotherapy response between different risk groups and found that the TIDE score was higher in the high-risk group of TCGA-LUAD than in the low-risk group (**Figure 5A**). Subsequently, we analyzed the expressions of immune checkpoints in the two risk groups and observed that CD274, PDCD1, and LAG3 were highly expressed in the high-risk group, indicating a higher likelihood of immune escape in this group (**Figure 5B**).

### Single-cell atlas of LUAD

**Figure 6:**
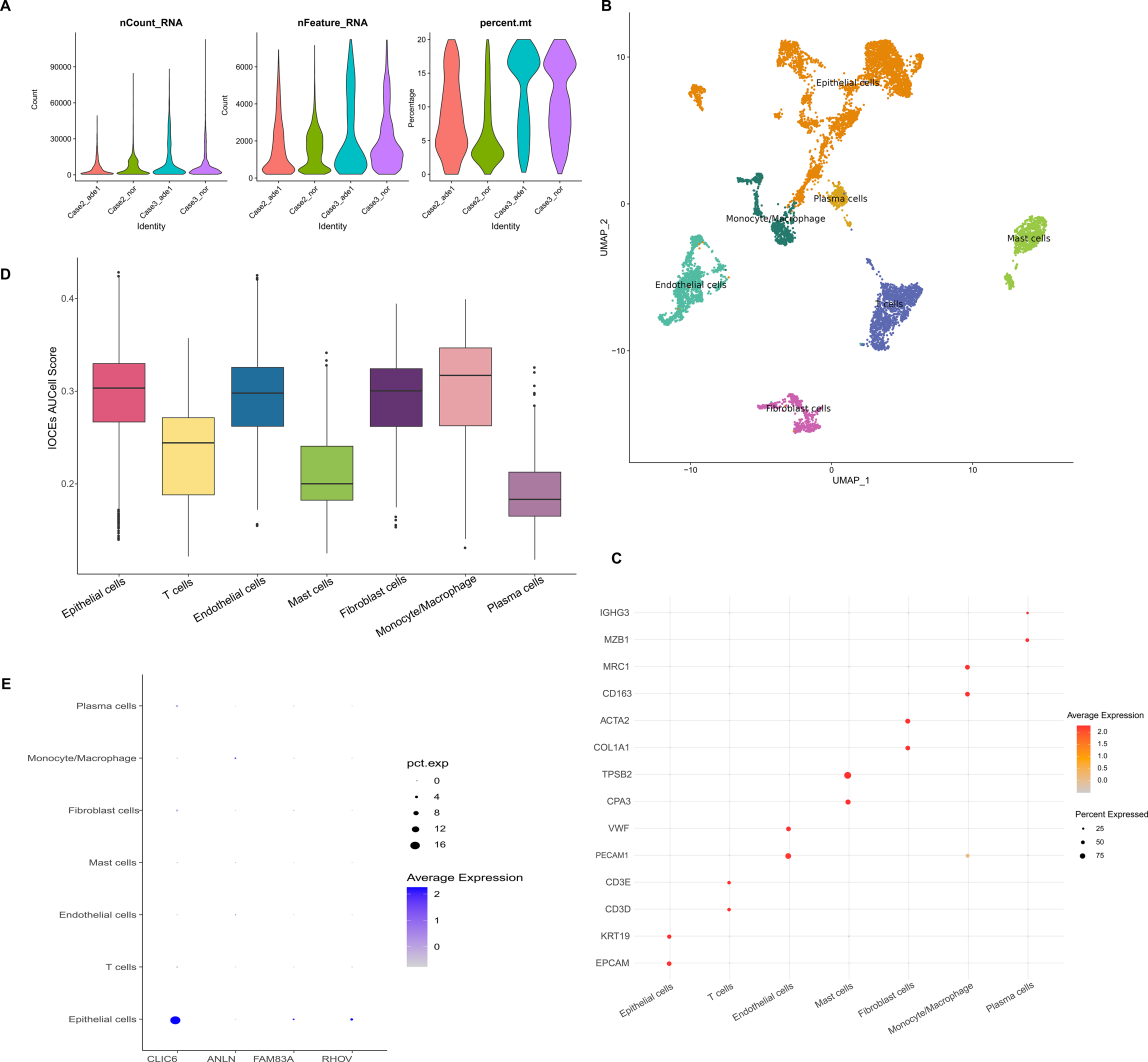
LUAD single-cell atlas. **(A)** LUAD single-cell transcriptomic quality control violin plot. **(B)** LUAD single-cell clustering and annotation UMAP dimensionality reduction plot. **(C)** Bubble plot showing marker gene expression for annotation of each cell subpopulation. **(D)** Differences in AUCell scores related to exosomes. **(E)** Expression of LUAD prognosis-related genes in each cell subpopulation.

The GSE149655 dataset contains two tumor lesion samples and two control samples. When performing single-cell transcriptomics analysis, we retained high-quality cells with a mitochondrial gene proportion of less than 20% and a detected gene count between 200 and 7,500 (**Figure 6A**). Following clustering analysis and annotation, seven cell subpopulations were identified, including: Endothelial cells, Monocyte/Macrophage, Epithelial cells, Mast cells, Fibroblast cells, Plasma cells, and T cells (**Figure 6B**). The marker genes for each cell subpopulation are shown in **Figure 6C**. Furthermore, we assessed the enrichment activity of exosome-related pathways in tumor samples and adjacent non-tumor samples, and the results indicated that the enrichment scores were higher in Epithelial cells and Monocyte/Macrophage cells than in other cell types (**Figure 6D**). Additionally, analysis of the expression patterns of prognostic-related genes revealed that CLIC6 was highly expressed in Epithelial cells (**Figure 6E**).

### Regulatory role of biomarkers at the cancer cell level

**Figure 7:**
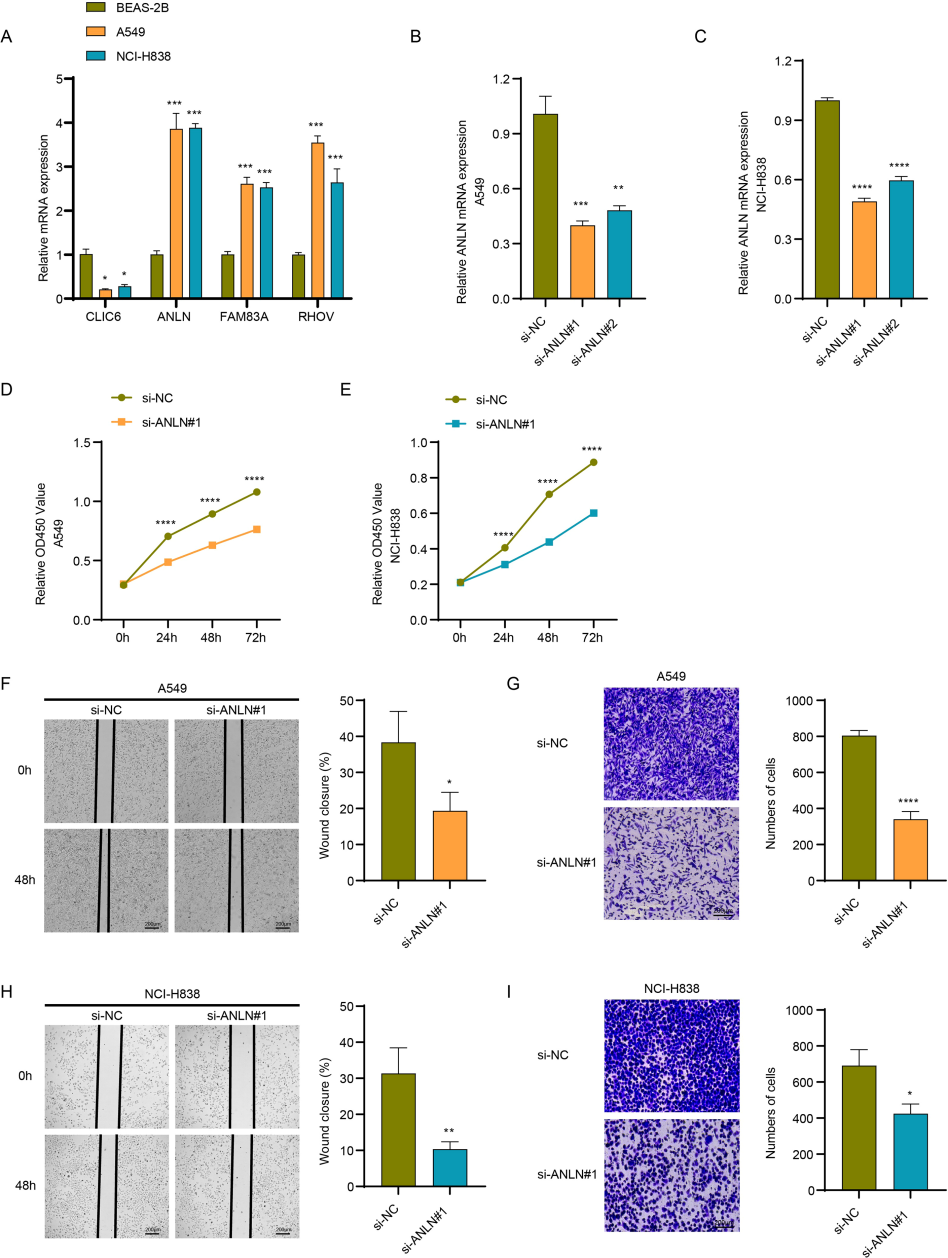
Regulatory effects of LUAD biomarkers on cancer cells. **(A)** Relative expression levels of genes CLIC6, ANLN, FAM83A, and RHOV in A549 and NCI-H838 cells compared to BEAS-2B cells. **(B)** Construction of ANLN-silenced A549 cell line. **(C)** Construction of ANLN-silenced NCI-H838 cell line. **(D)** Relative proliferation levels of ANLN-silenced A549 cells measured by CCK-8 assay. **(E)** Relative proliferation levels of ANLN-silenced NCI-H838 cells measured by CCK-8 assay. **(F)** Relative migration levels of ANLN-silenced A549 cells assessed by scratch assay. **(G)** Transwell assay measuring relative invasion levels in ANLN-silenced A549 cell lines. **(H)** Wound healing assay measuring relative migration levels in ANLN-silenced NCI-H838 cell lines. **(I)** Transwell assay measuring relative invasion levels in ANLN-silenced NCI-H838 cell lines. ****p<0.0001, ***p<0.001, **p<0.01, *p<0.05.

This study measured the relative expression levels of multiple biomarkers in LUAD cell lines. We observed that ANLN expression was significantly higher in LUAD cell lines (A549 and NCI-H838) compared to BEAS-2B cells. Furthermore, its upregulation was most consistent and pronounced across both cancer cell types, suggesting that ANLN may play a widespread and critical oncogenic role in LUAD. Consequently, we selected it for subsequent functional validation (**Figure 7A**). We established lung cancer cell lines A549/si-ANLN and NCI-H838/si-ANLN to achieve ANLN gene silencing (**Figures 7B, C**). CCK-8 assays demonstrated that ANLN silencing significantly inhibited the proliferation capacity of lung cancer cell lines (**Figures 7D, E**). Transwell and scratch assays revealed that ANLN silencing markedly suppressed the migration and invasion capabilities of lung cancer cell lines (**Figures 7F–I**).

## Discussion

Exosomes are major regulators of cellular signaling, coordinating various autocrine and paracrine functions, influencing intercellular communication, and thereby altering the tumor microenvironment, growth, and progression (32, 33). Previous studies have proposed diagnostic models for non-small cell lung cancer based on exosome-associated genes (34). Yuan et al. further applied exosome-associated RNA networks for molecular classification in LUAD prognosis assessment, identifying the SNHG6-hsa-miR-429-CHRDL1/CCNA2 axis as a potential novel therapeutic target for LUAD (35). Against the backdrop of the aforementioned research, this study further expands the clinical application scope of exosome-associated genes in LUAD. Its innovation lies primarily in the systematic integration of multi-omics data to construct and validate a prognostic risk model based on exosome-associated genes. The core finding of this study is the identification of four key exosome-associated genes (including CLIC6, ANLN, FAM83A, and RHOV) and the establishment of a model using these genes that effectively distinguishes patients’ prognostic risk levels. This reveals that the high-risk group exhibits significant immunosuppressive microenvironment characteristics and poorer potential for immune therapy response. Crucially, *in vitro* functional experiments confirmed ANLN’s distinct oncogenic role in LUAD, providing direct evidence for its potential as a therapeutic target. The potential value of this study lies not only in providing new biomarker tools for LUAD prognosis assessment but also in establishing a theoretical basis and experimental support for identifying immunotherapy-insensitive populations and developing therapeutic strategies targeting exosomal signaling pathways by correlating immune characteristics with treatment response. This research holds promise for advancing personalized LUAD treatment in clinical practice.

The genes identified in this study that are associated with LUAD progression and exosome characteristics include CLIC6, ANLN, FAM83A, and RHOV. Recent studies suggest that the chloride channel protein CLIC6 may play a key regulatory role in cancer development, as evidenced by its involvement in ion transport and cellular signaling, as well as its role in regulating multiple cellular functions and the tumor microenvironment (36). Notably, CLIC6 is downregulated in hepatocellular carcinoma and is significantly associated with the infiltration levels of six distinct immune cell types (macrophages, dendritic cells, neutrophils, CD8+ T cells, B cells, CD4+ T cells) (37). This has implications for the present study, as it similarly revealed a significant correlation between Riskscore and multiple immune cell types in LUAD, including T cells and B lineage cells. ANLN has been reported as a key regulatory factor in lung cancer metastasis and growth. Specifically, patients with high ANLN expression exhibit significantly more metastasis than those with low ANLN expression, and silencing ANLN significantly downregulates the migration and invasion capabilities of LUAD cell lines (38, 39). This further validates the efficacy of ANLN as a potential biomarker for LUAD in this study. FAM83A is also recognized as a poor prognostic factor in lung cancer, and its knockout inhibits LUAD cell proliferation, migration, and invasion (40). Therefore, the high expression of this gene in LUAD has also attracted widespread attention. Related studies have used proteomics and other research methods to clarify that FAM83A can maintain the stem cell-like phenotype of LUAD cells by stabilizing the human epidermal growth factor receptor, thereby promoting LUAD progression (41). RHOV, as a prognostic risk factor in LUAD in this study, aligns with existing research findings, which indicate that RHOV overexpression promotes the malignant phenotype of LUAD cell lines (42). In terms of regulatory mechanisms, this gene promotes the metastatic phenotype of LUAD cell lines through the JNK/c-Jun pathway, which is closely related to EGFR-TKI resistance in LUAD patients (42, 43). The genes identified in this study based on exosome characteristics are related to LUAD progression and hold significant value for LUAD prognosis assessment.

This study further explores the role of risk models in assessing immune responses in LUAD to elucidate the immune regulatory mechanisms underlying LUAD. In LUAD samples, patients with high Riskscore exhibited significantly elevated TIDE scores, suggesting that the high-risk group is more prone to activating tumor immune escape mechanisms, thereby reducing the potential benefits of immune checkpoint inhibitor therapy. The TIDE algorithm achieves high accuracy in predicting patient response to PD-1/PD-L1 and CTLA-4 inhibitors. As previously reported, high TIDE scores are closely associated with poor ICI treatment outcomes and shorter post-treatment survival (44, 45). Further analysis revealed that the Exclusion score in the high-risk group of LUAD was significantly higher than that in the low-risk group, reflecting impaired T cell infiltration into the tumor, which is associated with the immunosuppressive microenvironment composed of tumor-associated macrophages and tumor-associated fibroblasts, as previously reported (46, 47). Additionally, the high-risk group exhibited significantly upregulated expression of immune checkpoint genes CD274 (PD-L1), PDCD1 (PD-1), and LAG3, a feature consistent with the mechanism by which tumors evade immune surveillance by enhancing immunosuppressive signals. Related studies have shown that high expression of PD-1/PD-L1 is associated with resistance to immune checkpoint inhibitor therapy, while LAG3, another important immune inhibitory receptor, is also a marker of tumor immune tolerance and immune escape when highly expressed (48, 49). These results collectively indicate that high-risk LUAD patients assessed using the Riskscore in this study possess multiple immune escape mechanisms, making them unlikely to benefit from immunotherapy.

While this study provides insights into exosome-associated prognostic markers in LUAD, several limitations should be acknowledged. First, the single-cell transcriptomic analysis was based on a relatively small sample size, which may affect the generalizability of the identified cell subpopulations. Future studies should expand the cohort size and validate cellular composition using independent single-cell datasets. Second, the prognostic model and exosome-related genes were derived primarily from public databases and have not been validated at the tissue level or in animal models. Further immunohistochemical staining and *in vivo* functional studies are necessary to confirm their biological and clinical relevance. Third, although ANLN was experimentally validated, the roles of other key genes (including CLIC6, FAM83A, RHOV) in LUAD progression and immune regulation remain to be elucidated. Mechanistic studies focusing on these genes will help clarify their functional networks. Finally, the clinical utility of the risk model requires validation in prospective, multi-center cohorts before translation into practice. Overall, addressing these limitations in future work will enhance the robustness of our findings and support the development of exosome-targeted therapeutic strategies for LUAD.

## Conclusion

This study identified genes CLIC6, ANLN, FAM83A, RHOV as exosome-related prognostic genes, and established a risk model for LUAD. This model classifies patients into prognostic groups and is related to the level of immune infiltration and immunotherapy efficacy. Functional validations assays verified that ANLN may play an oncogenic role. These results could provide a useful risk assessment tool and highlight the potential therapeutic targets of the findings. These could assist in personalizing treatment, particularly the selection of high-risk patients for closer monitoring or immunotherapy.

## Data Availability

The datasets presented in this study can be found in online repositories. The names of the repository/repositories and accession number(s) can be found in the article/**Supplementary Material**.

## References

[1] Shi J Chen Y Peng C Kuang L Zhang Z Li Y . Advances in targeted therapy against driver mutations and epigenetic alterations in non-small cell lung cancer. Oncologie. (2022) 24:613–48. doi: 10.32604/oncologie.2022.027545, PMID: 40612875

[2] Xu L Li K Li J Liu L Xu F Xu Y . MiR-21/sonic hedgehog (SHH)/PI3K/AKT pathway is associated with NSCLC of primary EGFR-TKI resistance. Oncologie. (2022) 24:579–90. doi: 10.32604/oncologie.2022.022121, PMID: 40612875

[3] Cai Y Sheng Z Dong Z Wang J . EGFR inhibitor CL-387785 suppresses the progression of lung adenocarcinoma. Curr Mol Pharmacol. (2023) 16:211–6. doi: 10.2174/1874467215666220329212300, PMID: 35352671

[4] Succony L Rassl DM Barker AP McCaughan FM Rintoul RC . Adenocarcinoma spectrum lesions of the lung: Detection, pathology and treatment strategies. Cancer Treat Rev. (2021) 99:102237. doi: 10.1016/j.ctrv.2021.102237, PMID: 34182217

[5] Garrido J Bernal Y Gonzalez E Blanco A Sepulveda-Hermosilla G Freire M . Beyond tobacco: genomic disparities in lung cancer between smokers and never-smokers. BMC Cancer. (2024) 24:951. doi: 10.1186/s12885-024-12737-1, PMID: 39097719 PMC11297669

[6] Ruiz-Cordero R Devine WP . Targeted therapy and checkpoint immunotherapy in lung cancer. Surg Pathol Clin. (2020) 13:17–33. doi: 10.1016/j.path.2019.11.002, PMID: 32005431

[7] Sun Y Zhang Y Ren S Li X Yang P Zhu J . Low expression of RGL4 is associated with a poor prognosis and immune infiltration in lung adenocarcinoma patients. Int Immunopharmacol. (2020) 83:106454. doi: 10.1016/j.intimp.2020.106454, PMID: 32259700

[8] Pozza DH Andrade de Mello RB . Treatment sequencing strategies in lung cancer. Zhongguo Fei Ai Za Zhi. (2022) 25:323–36. doi: 10.3779/j.issn.1009-3419.2022.104.01, PMID: 35599008 PMC9127753

[9] Schuler M Bolukbas S Darwiche K Theegarten D Herrmann K Stuschke M . Personalized treatment for patients with lung cancer. Dtsch Arztebl Int. (2023) 120:300–10. doi: 10.3238/arztebl.m2023.0012, PMID: 36790172 PMC10391522

[10] Wu Q Wang L Wei H Li B Yang J Wang Z . Integration of multiple key molecules in lung adenocarcinoma identifies prognostic and immunotherapeutic relevant gene signatures. Int Immunopharmacol. (2020) 83:106477. doi: 10.1016/j.intimp.2020.106477, PMID: 32278127

[11] Saad FA . Precision medicine: design of immune inert exosomes for targeted gene delivery. Curr Gene Ther. (2025) 25. doi: 10.2174/0115665232286489240320051925, PMID: 41031497

[12] Zhao L Gu C Gan Y Shao L Chen H Zhu H . Exosome-mediated siRNA delivery to suppress postoperative breast cancer metastasis. J Controlled Release: Off J Controlled Release Soc. (2020) 318:1–15. doi: 10.1016/j.jconrel.2019.12.005, PMID: 31830541

[13] Zhao Y Zheng Y Zhu Y Zhang Y Zhu H Liu T . M1 macrophage-derived exosomes loaded with gemcitabine and deferasirox against chemoresistant pancreatic cancer. Pharmaceutics. (2021) 13:1493. doi: 10.3390/pharmaceutics13091493, PMID: 34575569 PMC8472397

[14] Li J Peng J Wang J Chen Z . The dual role of exosomes in the tumor microenvironment: from pro-tumorigenic signaling to immune modulation. Med Res. (2025) 1:257–84. doi: 10.1002/mdr2.70022, PMID: 41848424

[15] Peng J Liang Q Xu Z Cai Y Peng B Li J . Current understanding of exosomal microRNAs in glioma immune regulation and therapeutic responses. Front Immunol. (2021) 12:813747. doi: 10.3389/fimmu.2021.813747, PMID: 35095909 PMC8796999

[16] Peyruchaud O Saier L Leblanc R . Autotaxin implication in cancer metastasis and autoimunne disorders: functional implication of binding autotaxin to the cell surface. Cancers (Basel). (2019) 12:105. doi: 10.3390/cancers12010105, PMID: 31906151 PMC7016970

[17] Shah DR Masters GA . Precision medicine in lung cancer treatment. Surg Oncol Clin N Am. (2020) 29:15–21. doi: 10.1016/j.soc.2019.08.002, PMID: 31757310

[18] Zhang L Yu D . Exosomes in cancer development, metastasis, and immunity. Biochim Biophys Acta Rev Cancer. (2019) 1871:455–68. doi: 10.1016/j.bbcan.2019.04.004, PMID: 31047959 PMC6542596

[19] Ma H Jiang B Ren Q Sun Y Wang M Xia S . Exosomal miR-20b-5p induces EMT and enhances progression in non-small cell lung cancer via TGFBR2 downregulation. J Biochem Mol Toxicol. (2024) 38:e70080. doi: 10.1002/jbt.70080, PMID: 39635830

[20] Huang L Wang F Wang X Su C Wu S Yang C . M2-like macrophage-derived exosomes facilitate metastasis in non-small-cell lung cancer by delivering integrin alphaVbeta3. MedComm. (2020) 4:e191. doi: 10.1002/mco2.191, PMID: 36582304 PMC9789322

[21] Morrissey SM Zhang F Ding C Montoya-Durango DE Hu X Yang C . Tumor-derived exosomes drive immunosuppressive macrophages in a pre-metastatic niche through glycolytic dominant metabolic reprogramming. Cell Metab. (2021) 33:2040–2058.e10. doi: 10.1016/j.cmet.2021.09.002, PMID: 34559989 PMC8506837

[22] He Z Wang J Zhu C Xu J Chen P Jiang X . Exosome-derived FGD5-AS1 promotes tumor-associated macrophage M2 polarization-mediated pancreatic cancer cell proliferation and metastasis. Cancer Lett. (2022) 548:215751. doi: 10.1016/j.canlet.2022.215751, PMID: 35718269

[23] Song Z Yu J Wang M Shen W Wang C Lu T . CHDTEPDB: transcriptome expression profile database and interactive analysis platform for congenital heart disease. Congenital Heart Dis. (2023) 18:693–701. doi: 10.32604/chd.2024.048081, PMID: 40612875

[24] Ritchie ME Phipson B Wu D Hu Y Law CW Shi W . limma powers differential expression analyses for RNA-sequencing and microarray studies. Nucleic Acids Res. (2015) 43:e47. doi: 10.1093/nar/gkv007, PMID: 25605792 PMC4402510

[25] Wilkerson MD Hayes DN . ConsensusClusterPlus: a class discovery tool with confidence assessments and item tracking. Bioinformatics. (2010) 26:1572–3. doi: 10.1093/bioinformatics/btq170, PMID: 20427518 PMC2881355

[26] Simon N Friedman J Hastie T Tibshirani R . Regularization paths for cox’s proportional hazards model via coordinate descent. J Stat Softw. (2011) 39:1–13. doi: 10.18637/jss.v039.i05, PMID: 27065756 PMC4824408

[27] Blanche P Dartigues JF Jacqmin-Gadda H . Estimating and comparing time-dependent areas under receiver operating characteristic curves for censored event times with competing risks. Stat Med. (2013) 32:5381–97. doi: 10.1002/sim.5958, PMID: 24027076

[28] Becht E Giraldo NA Lacroix L Buttard B Elarouci N Petitprez F . Estimating the population abundance of tissue-infiltrating immune and stromal cell populations using gene expression. Genome Biol. (2016) 17:218. doi: 10.1186/s13059-016-1070-5, PMID: 27765066 PMC5073889

[29] Stuart T Butler A Hoffman P Hafemeister C Papalexi E Mauck WM 3rd . comprehensive integration of single-cell data. Cell. (2019) 177:1888–1902.e21. doi: 10.1016/j.cell.2019.05.031, PMID: 31178118 PMC6687398

[30] Korsunsky I Millard N Fan J Slowikowski K Zhang F Wei K . Fast, sensitive and accurate integration of single-cell data with Harmony. Nat Methods. (2019) 16:1289–96. doi: 10.1038/s41592-019-0619-0, PMID: 31740819 PMC6884693

[31] Aibar S González-Blas CB Moerman T Huynh-Thu VA Imrichova H Hulselmans G . SCENIC: single-cell regulatory network inference and clustering. Nat Methods. (2017) 14:1083–6. doi: 10.1038/nmeth.4463, PMID: 28991892 PMC5937676

[32] Sung BH von Lersner A Guerrero J Krystofiak ES Inman D Pelletier R . A live cell reporter of exosome secretion and uptake reveals pathfinding behavior of migrating cells. Nat Commun. (2020) 11:2092. doi: 10.1038/s41467-020-15747-2, PMID: 32350252 PMC7190671

[33] Zhao Y Liu T Zhou M . Immune-cell-derived exosomes for cancer therapy. Mol Pharm. (2022) 19:3042–56. doi: 10.1021/acs.molpharmaceut.2c00407, PMID: 35876318

[34] Sun Z Du T Yang G Sun Y Xiao X . Identification of exosome-related genes in NSCLC via integrated bioinformatics and machine learning analysis. Sci Rep. (2025) 15:22962. doi: 10.1038/s41598-025-04485-4, PMID: 40595797 PMC12215682

[35] Yuan Y Li Q Chen F Zhao Y Ma J Fang S . Identification of an exosome-relevant SNHG6-hsa-miR-429- CHRDL1/CCNA2 axis for lung adenocarcinoma prognosis evaluation. Curr Med Chem. (2024) 31:4549–61. doi: 10.2174/0109298673280925231122104717, PMID: 38994652

[36] Shu L Tang J Liu S Tao Y . Plasma cell signatures predict prognosis and treatment efficacy for lung adenocarcinoma. Cell Oncol (Dordr). (2024) 47:555–71. doi: 10.1007/s13402-023-00883-w, PMID: 37814076 PMC12973951

[37] Huang JJ Lin J Chen X Zhu W . Identification of chloride intracellular channels as prognostic factors correlated with immune infiltration in hepatocellular carcinoma using bioinformatics analysis. Med (Baltimore). (2021) 100:e27739. doi: 10.1097/MD.0000000000027739, PMID: 34766585 PMC10545300

[38] Xu J Zheng H Yuan S Zhou B Zhao W Pan Y . Overexpression of ANLN in lung adenocarcinoma is associated with metastasis. Thorac Cancer. (2019) 10:1702–9. doi: 10.1111/1759-7714.13135, PMID: 31268619 PMC6669805

[39] Sheng L Kang Y Chen D Shi L . Knockdown of ANLN inhibits the progression of lung adenocarcinoma via pyroptosis activation. Mol Med Rep. (2023) 28:177. doi: 10.3892/mmr.2023.13064, PMID: 37539739 PMC10433705

[40] Wang G Li X Yao Y Jiang Z Zhou H Xie K . FAM83A and FAM83A-AS1 both play oncogenic roles in lung adenocarcinoma. Oncol Lett. (2021) 21:297. doi: 10.3892/ol.2021.12558, PMID: 33732373 PMC7905536

[41] Yuan Y Hao L Huang JS Zhao FY Ju YH Wang JM . Promotion of stem cell-like phenotype of lung adenocarcinoma by FAM83A via stabilization of ErbB2. Cell Death Dis. (2024) 15:460. doi: 10.1038/s41419-024-06853-w, PMID: 38942760 PMC11213963

[42] Chen H Xia R Jiang L Zhou Y Xu H Peng W . Overexpression of rhoV promotes the progression and EGFR-TKI resistance of lung adenocarcinoma. Front Oncol. (2021) 11:619013. doi: 10.3389/fonc.2021.619013, PMID: 33767988 PMC7986718

[43] Zhang D Jiang Q Ge X Shi Y Ye T Mi Y . RHOV promotes lung adenocarcinoma cell growth and metastasis through JNK/c-Jun pathway. Int J Biol Sci. (2021) 17:2622–32. doi: 10.7150/ijbs.59939, PMID: 34326698 PMC8315012

[44] Huang X Liu Y Qian C Shen Q Wu M Zhu B . CHSY3 promotes proliferation and migration in gastric cancer and is associated with immune infiltration. J Transl Med. (2023) 21:474. doi: 10.1186/s12967-023-04333-x, PMID: 37461041 PMC10351153

[45] Wang F Lin H Su Q Li C . Cuproptosis-related lncRNA predict prognosis and immune response of lung adenocarcinoma. World J Surg Oncol. (2022) 20:275. doi: 10.1186/s12957-022-02727-7, PMID: 36050740 PMC9434888

[46] Fang W Zhou T Shi H Yao M Zhang D Qian H . Progranulin induces immune escape in breast cancer via up-regulating PD-L1 expression on tumor-associated macrophages (TAMs) and promoting CD8(+) T cell exclusion. J Exp Clin Cancer Res. (2021) 40:4. doi: 10.1186/s13046-020-01786-6, PMID: 33390170 PMC7780622

[47] Arpinati L Scherz-Shouval R . From gatekeepers to providers: regulation of immune functions by cancer-associated fibroblasts. Trends Cancer. (2023) 9:421–43. doi: 10.1016/j.trecan.2023.01.007, PMID: 36870916

[48] Tang Q Chen Y Li X Long S Shi Y Yu Y . The role of PD-1/PD-L1 and application of immune-checkpoint inhibitors in human cancers. Front Immunol. (2022) 13:964442. doi: 10.3389/fimmu.2022.964442, PMID: 36177034 PMC9513184

[49] Andrews LP Marciscano AE Drake CG Vignali DA . LAG3 (CD223) as a cancer immunotherapy target. Immunol Rev. (2017) 276:80–96. doi: 10.1111/imr.12519, PMID: 28258692 PMC5338468

