# Supplementary material for: CLIC6 and ANLN: novel exosome-related prognostic markers and therapeutic targets in lung adenocarcinoma
Source: Front Immunol. 2026 Mar 23;17:1756058. doi: 10.3389/fimmu.2026.1756058 (PMC13050664; doi:10.3389/fimmu.2026.1756058)
Supplement: Supplementary file 2 [file DataSheet1.docx]

The raw data is available at Jianguoyun link: https://www.jianguoyun.com/p/DfvjN4IQwcXwDRjL8pMGIAA.
